# Vision is protected against blue defocus

**DOI:** 10.1038/s41598-020-79911-w

**Published:** 2021-01-11

**Authors:** Clara Benedi-Garcia, Maria Vinas, Carlos Dorronsoro, Stephen A. Burns, Eli Peli, Susana Marcos

**Affiliations:** 1 Instituto de Óptica Daza de Valdés, Consejo Superior de Investigaciones Científicas (IO-CSIC), Madrid, Spain; 2 School of Optometry, Indiana University, Bloomington, IN USA; 3 The Schepens Eye Research Institute-Massachusetts Eye and Ear Infirmary, Department of Ophthalmology, Harvard Medical School, Boston, MA USA

**Keywords:** Applied optics, Adaptive optics

## Abstract

Due to chromatic aberration, blue images are defocused when the eye is focused to the middle of the visible spectrum, yet we normally are not aware of chromatic blur. The eye suffers from monochromatic aberrations which degrade the optical quality of all images projected on the retina. The combination of monochromatic and chromatic aberrations is not additive and these aberrations may interact to improve image quality. Using Adaptive Optics, we investigated the optical and visual effects of correcting monochromatic aberrations when viewing polychromatic grayscale, green, and blue images. Correcting the eye’s monochromatic aberrations improved optical quality of the focused green images and degraded the optical quality of defocused blue images, particularly in eyes with higher amounts of monochromatic aberrations. Perceptual judgments of image quality tracked the optical findings, but the perceptual impact of the monochromatic aberrations correction was smaller than the optical predictions. The visual system appears to be adapted to the blur produced by the native monochromatic aberrations, and possibly to defocus in blue.

## Introduction

The human eye’s optical imperfections (known as aberrations) blur images projected onto the retina. In addition to aberrations that occur for stimuli in all colors, known as monochromatic aberrations, the refractive index of the ocular media varies with wavelength. This variation causes the focal power of the eye to vary by almost 2 diopters (D) across the visible spectrum, an effect known as longitudinal chromatic aberration (LCA)^1^.

Despite the degradation of the optical quality caused by Higher Order Aberrations (HOAs), observers are not aware of the blur present in their retinal images, reflecting both the sampling properties of retinal neurons and the underlying neural adaptation to the native optical blur^2^. This neural adaptation, a recalibration process in the visual system that diminishes the impact of retinal images blur, is ubiquitous in vision, as the visual system adapts to changes in the optics and the environment over time, using similar strategies across multiple stimulus domains (color, contrast, spatial frequency, or face perception)^3^.

The defocus of the retinal image caused by the LCA of the eye is potentially high. The chromatic defocus between the wavelength of the maximum sensitivity of S and M/L cones is ~ 1.5 D, equivalent to a ~ 26 arcmin blur circle for a 5 mm pupil^4^. Yet, the visual system is sensitive to blur as small as 1 arcmin, although this is dependent on the context of the blur^5^. An important unresolved question is why perception of images is not severely degraded by the chromatic defocus? While the visual impact of the LCA on polychromatic image quality is reduced by the spectral sensitivity of the retinal photoreceptors^1,6–8^, the effect of LCA blurring on monochromatic targets when the eye is focused in the middle of the spectrum is large. Understanding how the visual system copes with LCA is relevant to many visual processes^9^. For instance, chromatic aberration has often been invoked as a polarity cue for eye growth during emmetropization and for controlling the response of the crystalline lens during accommodation^10,11^. The process of adaptation to chromatic blur may also occur during cataract development and may play a role in the adaptation to replacement of the eye’s lens with an intraocular lens (IOL) following cataract surgery, since the magnitude of chromatic aberration of the crystalline lens and IOL differ^12,13^. With the development of new diffractive multifocal IOL designs, it is also possible to modulate chromatic aberration independently for the far, intermediate or near foci, canceling LCA for at least some distances^14^. However, the visual impact of removing LCA remains an open question^10^.

The relatively lower optical contribution of LCA to perceived image quality, which is theoretically largest at short wavelengths, is traditionally attributed to two factors: the paucity of S-cones in the central fovea and the absorption of short wavelength light by the yellowish macular pigment. Two decades ago, some of the authors of the current study reported that invoking those mechanisms may not be necessary, and that the magnitude of the effect of the latter was in fact minimal^15^. Instead, it was found that, while the optical contrast reduction produced by LCA in S-cones with respect to M/L cones was large in diffraction-limited eyes (free of monochromatic aberrations), the presence of the eye’s natural monochromatic aberrations mitigated the differential impact of LCA on optical contrast for S-cones and M/L cones. In fact, optical simulations showed that the combination of natural monochromatic and chromatic aberrations boosted contrast for blue stimuli when the eye was focused in the green compared to an aberration-corrected eye. Aberrated optics therefore seemed to provide the eye partial protection against chromatic blur.

Favorable interaction between monochromatic aberrations and defocus have been shown to occur, and these interactions can improve the eye’s modulation transfer function out of focus^16^ increasing the depth-of-focus of the eye^17–19^. Depth-of-focus has also been invoked as a factor important in the evolution of visual pigments and trichromatic color vision^20^. However, the potential perceptual consequences of the optical interactions between LCA and high order monochromatic aberrations have not been investigated.

Adaptive Optics (AO), is used to compensate for the monochromatic aberrations of the eye, both in fundus imaging and in psychophysical experiments^21^. AO has also provided a tool for probing neural adaptation to new aberration patterns^2,22,23^. Thus, AO enables testing the hypothesis that increased depth-of-focus arising from the optical interactions of LCA and monochromatic aberrations can account for the relative insensitivity of human vision to chromatic blur in the short wavelength components of a polychromatic image. If this is not the case, other perceptual (adaptation) mechanisms may play controlling roles. The question is timely as intraocular lens (IOL) manufacturers embark on developing new designs to reduce LCA. These developments will lead to patients fitted with IOLs that alter the balance of monochromatic/polychromatic aberrations, which may be needed to perceptually recalibrate to a new spatial/chromatic environment.

## Methods

An Adaptive Optics system consisting on an optical channel to measure and correct the optical aberrations of the eye and a psychophysical channel to present visual stimuli (monochromatic and polychromatic blue or green stimuli, as well as broadband gray scale) was used. Observers viewed stimuli with natural (NoAO) or fully corrected (AO) monochromatic aberrations, in three experiments that evaluated the visual effect of chromatic defocus.

### Subjects

Ten subjects with normal vision participated in the study (ages ranging from 22 to 45 years old, (31 ± 9); average spherical error: − 1.23 ± 2.28 D, cylinder: − 0.20 ± 0.23 D). The pupil was dilated and the accommodation was paralyzed using Tropicamide 1% (2 drops instilled at the beginning of the session and re-instilled every hour). The study was explained to the subjects, before they were asked to sign informed consents. The study met the tenets of the Declaration of Helsinki, and the protocols were approved by the CSIC Institutional Review Board.

### Adaptive optics system

**Figure 1:**
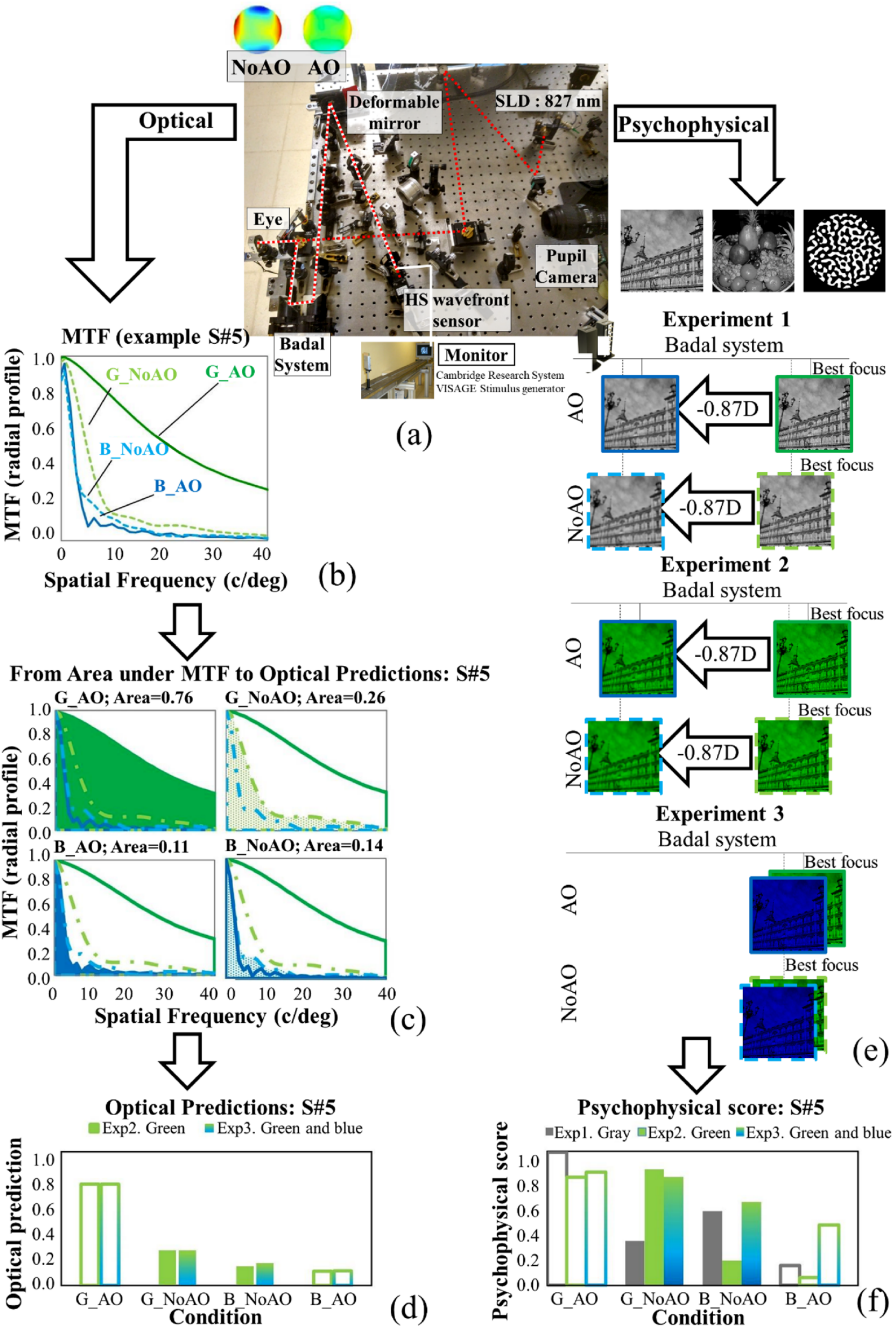
Methods overview. Example of Optical predictions (left) and Psychophysical scores (right) for one subject (S#5) from experimental measurements in the Adaptive Optics system (with natural and AO-corrected aberrations, depicted in (**a**)). The optical modulation transfer functions (MTFs) were obtained from the measured wave aberrations at best focus for Green (NoAO, dotted line and AO, solid line) and for a defocus corresponding to the chromatic difference of focus for Blue (**b**). Optical quality predictions are estimated computing the normalized area under the MTF (0–40 c/deg range) (**c**,**d**). The patients then scores the perceived quality of natural images (city scene, fruit platter and binary noise) of gray-scale images (in focus, and defocus by − 0.87 D), green images (in focus, and defocus by − 0.87 D), and green and blue images (in the best focus of green), both with natural aberrations (NoAO) and aberration correction (AO), illustrated in (**e**). Psychophysical scores are obtained from the perceptual judgment of natural images (0–5) as seen through the Adaptive Optics system in 4 conditions (G_AO, G_NoAO, B_NoAO) and normalized to 1 (**f**). Note that G_AO and G_NoAO means best focus of gray-scale images in Exp 1 and best focus for green in Experiment 2 and 3, for AO and NoAO conditions, respectively, and B_AO and B_NoAO means − 0.87 D from the best focus of gray-scale images and green images, for Experiment 1 and 2, respectively, and best focus of green (with blue stimuli) in Experiment 3.

Measurements were conducted in a custom Adaptive Optics (AO) system (Fig. 1a), described in detail previously^24^. The system uses a Super Luminescent Diode (λ = 827 mm, Superlum, Ireland) for illumination; a Hartmann-Shack wavefront sensor (32 × 32 microlenses; HASO 32 OEM, Imagine Eyes, France), and an electromagnetic deformable mirror (52 actuators of 50 µm stroke; MIRAO, Imagine Eyes, France), for measurement and correction of wave aberrations, respectively, placed at conjugate pupil planes of the system. A psychophysical channel, consisting of a CRT monitor (Mitsubishi Diamond Pro 2070, 2048 × 1536 pixels, 8-bit grayscale, 2-deg angular subtense) and a relay of lenses, projects the stimulus onto the subjects’ retina. A motorized Badal system compensated for spherical refractive errors, was adjusted for the best focus and also used to induce an equivalent chromatic defocus. The pupil monitoring channel included an LED ring illuminator and a CCD camera, and a 5-mm artificial pupil at a conjugate pupil plane.

Subjects were aligned to the optical set-up using a dental impression bite bar, aided by the pupil monitoring channel. Subjects adjusted subjectively their best focus for grayscale/green stimuli, which were previously blurred with positive defocus, using a Badal system. Subjects were asked to stop the first time that the stimulus seemed appeared in focus (method of limits). The subject’s aberrations were measured using the Hartmann-Shack, and corrected with the deformable mirror in a closed-loop procedure. Subjects adjusted subjectively their best focus also for the AO-corrected condition. The psychophysical measurements were performed under static correction of the mirror. Centration and the quality of the AO correction was checked regularly throughout the experiment. If the Root-Mean-Square Wavefront error increased above 0.1 µm, a new correction was measured and applied.

### Visual stimuli

Visual stimuli were natural images displayed on a CRT monitor: a city scene and a fruit platter as well as binary noise (Fig. 1e). These images were generated in polychromatic grayscale, green and blue versions. Stimuli’s size was 480 × 480 pixels. Colors were produced by canceling the other channels ((0, G, 0) for green and (0, 0, B) for blue). The emission of the CRT phosphors was characterized using a Minolta Spectroradiometer (CS-1000). Peaks were found at λ = 450 nm (56-nm bandwidth) for blue images and at λ = 550 nm (77-nm bandwidth) for green images. The CRT peak green luminance was 23 cd/m^2^. Mirrors and beam splitters produced 48% attenuation. Isoluminant green and blue stimuli were generated by adjusting the green color in a luminance matching psychophysical procedure.

### Psychophysical experiments

Subjects scored the perceived image quality of the visual stimuli under different conditions (grayscale, green and blue images; in and out of focus; with natural aberrations and with AO-correction), in three different experiments (Fig. 1e–f). The subjects were asked to score the images for their perceived visual quality according to their subjective perception on a scale from 0 to 5. Prior to scoring, sample images with in focus or out of focus were presented to the patient. From previous work^25^, the objectively sharpest images (full correction of aberrations) are not always judged as best (score of 5). Images were presented either at best focus or defocused by an amount equivalent to the chromatic difference of focus between green and blue (− 0.87 D)^26^. Three experiments were conducted:

- *Experiment 1* Grayscale images were viewed at the subject’s best subjective focus (selected separately under natural aberrations and under AO-correction), and defocused by − 0.87 D from best focus in each condition. In this experiment, we measured the effect of blue chromatic defocus level (induced as pure optical defocus) on multi-wavelength gray-scale images.
- *Experiment 2* Green images were viewed at the subject’s best subjective focus (under natural aberrations and AO-correction), and defocused by − 0.87 D from best focus in each condition. In this experiment, we measured the effect of blue chromatic defocus level (induced as pure optical defocus) on green images.
- *Experiment 3* Equi-luminant green and blue images were viewed at the subject’s best subjective focus obtained in green (under natural aberrations and under AO-correction), without shifting focus for blue (except for the shift resulting from the individual’s own chromatic aberration). In this experiment, we measured the effect of the natural chromatic defocus of blue images on blue and green images. The green images in this experiment are identical to those in experiment 2.

The three experiments were conducted in the same order for all subjects within a single experimental session, which typically lasted 2 h. In each experiment, images were presented to the subject for 1.5 s, and scoring of perceived image quality (a score of 0–5, on a keyboard) was made following an auditory cue. In each experiment, subjects scored the quality of 3 images, 9 repetitions in each condition. In each experiment, each condition consisted of a given focus position in the Badal system and a status of the deformable mirror. The condition of best focus in green or gray is labeled G. The conditions that were out-of-focus in gray or green, or blue with no-additional defocus in the Badal optometer is labeled as blue (B). The conditions in which the subject’s aberrations are corrected or left uncorrected are labeled AO or NoAO, respectively. Within a given stimulus condition, images were randomly interleaved and the quality judgements were made 9 times for each of the 3 images. Each condition consisted of 27 scores (3 images × 9 repetitions/image), and each experiment involved 108 trials (4 conditions × 27 scores/condition). The maximum possible score for each condition is 135 (27 scores × 5 maximum score). Total scores were normalized to 1 by dividing by 135.

### Control experiment using a narrow band AO set-up

A control experiment was performed in custom-developed polychromatic AO system, in which the stimuli were generated using narrow blue and green lines (spectral bandwidth of 5 nm) in place of the wider spectrum LED stimuli. Illumination was provided by a supercontinuum laser source (SCLS, SC400 femtopower 106 supercontinuum laser, Fianium Ltd, United Kingdom) that works in combination with a dual acousto-optic tunable filter (AOTF). The AO channel includes a Hartmann-Shack wavefront sensor (40 × 32 microlenses; HASO 32 OEM, Imagine Eyes, France) and an electromagnetic deformable mirror (52 actuators and 50 µm stroke; MIRAO, Imagine Eyes, France), placed at conjugate pupil planes of the system. The psychophysical channel consists of a Digital Micro-Mirror Device (DMD) (DLP (R) Discovery (TM) 4100 0.7 XGA, Texas Instruments, USA), which allows displaying visual stimuli (1.62° angular subtense) illuminated with the laser light. The coherence of the laser beam was scrambled using a holographic diffuser which provides a uniform illumination of the stimulus. A motorized Badal system is used to adjust for the best focus of the subjects as well as to induce the equivalent chromatic defocus. The eye’s pupil is monitored with an LED ring illuminator and a CCD camera. A 5-mm artificial pupil is placed at a conjugate pupil plane. A complete description of the polychromatic system with all its channels can be found in previous publications^26–28^. Two subjects from the original experiment (S2, high HOAs and S9, low HOAs) scored the same 3 images in three different conditions (corresponding to Experiments 2 and 3) under natural aberrations (NoAO): (1) Illumination with green light (λ = 550 nm) for the best subjective focus in green light; (2) Illumination with blue light (λ = 450 nm) for the best subjective focus in green light; (3) Illumination with green light (λ = 550 nm) for an induced defocus of − 0.87D from the best subjective focus in green light. As in the original experiment, the power of the green and blue laser beams were adjusted so that they produced equal perceived luminance. As for the main study, the experiment was performed with natural aberrations (NoAO) and with the aberrations corrected by the deformable mirror (AO).

### Optical predictions

Optical aberrations of each eye were measured before and after AO correction, and expressed by a Zernike polynomial expansion up to the 6th order, obtained as the average of 3 repeated measurements. Visual Strehl ratios (VS) were calculated from the measured wave aberrations (natural and AO-corrections) for 5-mm pupil diameters, for green (550 nm) and blue (450 nm) wavelengths (Fig. 1b). Since high order aberrations are largely independent of wavelength^9^, high order Zernike coefficients measured at 820 nm were used in the VS calculations. The wavelength was introduced as a scaling factor in the pupil function (both in the modulus and phase). Defocus was introduced in the phase of the pupil function as a defocus Zernike term. Chromatic defocus between 550 and 450 nm was assumed to be − 0.87 D^26^. VS was estimated as the normalized volume under the MTF (progressively truncated by the neural contrast threshold)^29,30^. Best focus in green was estimated as the defocus producing the largest VS. The optical quality prediction was estimated in all conditions replicating Experiments 2 and 3 (AO, NoAO, in focus and defocused by chromatic defocus, 5-mm pupil, Fig. 1c,d) normalized by the area under the MTF radial profile (0–40 c/° range).

### Statistical analysis

A one-way ANOVA test showed no statistical differences between perceptual scores assigned to the different images (noise, fruits and city) for a similar condition (p = 0.58), and therefore data was pooled across images.

To comparatively evaluate the weight of each effect, a Mixed Model Analysis with two fixed factors (condition and experiment), and subject as random factor in repeated measures was used for each type of result (optical and psychophysical). For each type of result (optical and psychophysical) and experiment we used a Mixed Model Analysis with one fixed factor (condition) and a random factor (subject).

## Results

### Ocular optical quality

**Figure 2:**
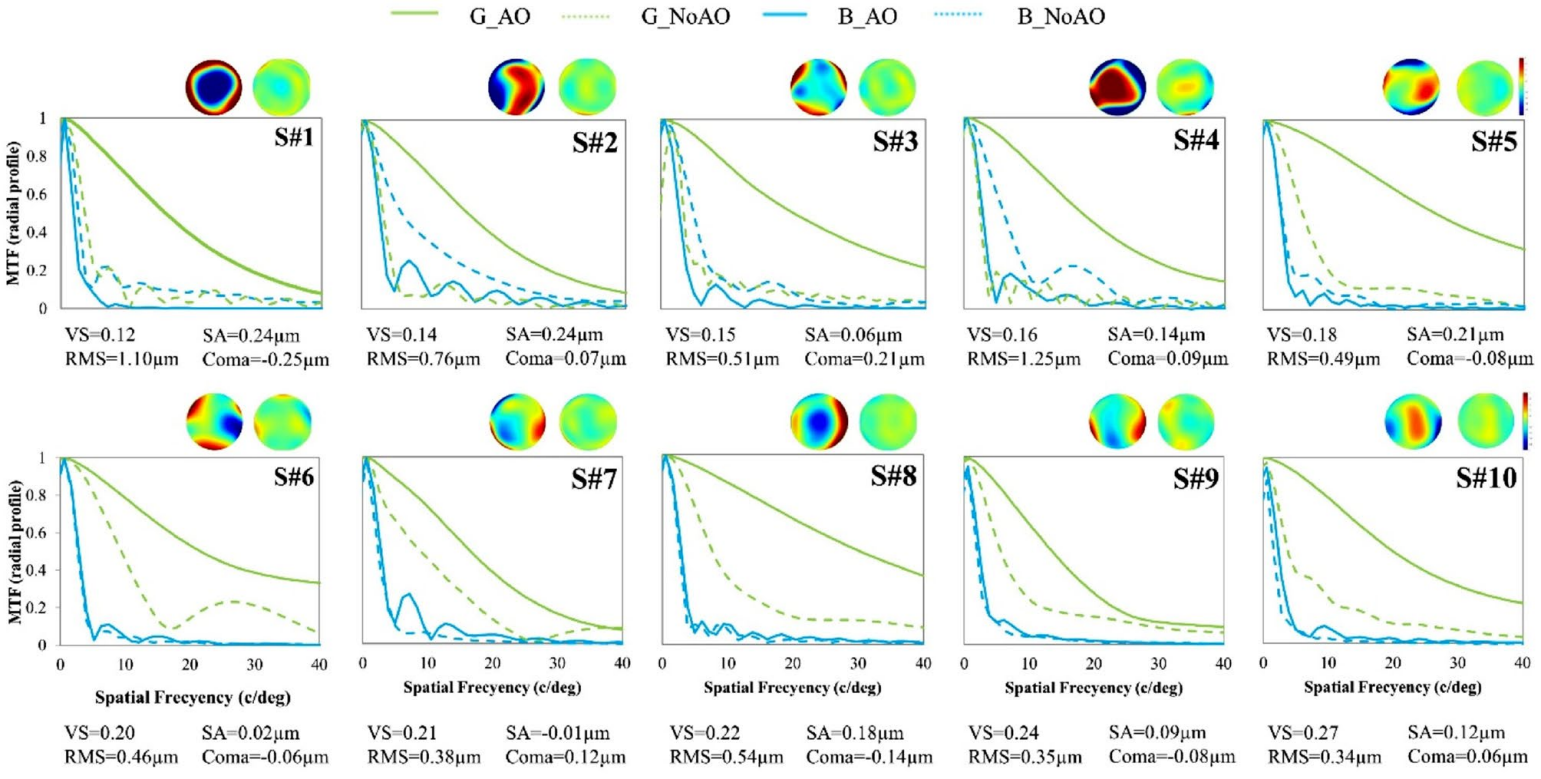
MTFs in all eyes, computed from the individually measured monochromatic aberrations. With natural aberrations (NoAO, dashed lines) and AO-corrected monochromatic aberrations (solid lines). MTFs are for best focus in green (G. 550 nm) and for the corresponding chromatic difference of focus (− 0.87 D, 450 nm) in blue. The maps above each graph represent monochromatic wave aberrations with no defocus and natural aberrations (left) and with AO-corrected aberration (right). Subjects are ordered according to increasing Visual Strehl (VS) value for 5-mm pupil diameters for the G_NoAO condition. Root Mean Square (RMS), Spherical Aberration (SA), and Coma values for NoAO correction (for the natural aberration condition) are shown in each panel under the subject’s label, unitless and μm units, respectively).

Figure 2 shows the optical quality for each measured eye in terms of the MTF computed from the wavefront sensor measurements. The MTF’s were radially averaged for green and blue light with natural aberrations (dotted lines: NoAO) and with adaptive optics correction of the monochromatic aberrations (solid lines: AO), calculated for 5-mm pupils. The colormaps on top of each graph show the corresponding wave aberrations at the pupil for NoAO (left) and AO (right). The MTFs in green are calculated for the focus which maximizes optical quality in green (both for NoAO and AO: G_NoAO and G_AO). The MTFs in blue are calculated for a chromatic defocus of − 0.87 D (450–550 nm)^26^ relative to the corresponding best focus in green (B_NoAO & B_AO). Subjects are labeled according to optical quality of their natural optics (G_NoAO), from lowest (S#1) to highest (S#10), in terms of Visual Strehl (VS^29^). Figure 2 represents the conditions tested in Experiment 2.

As expected, AO improved image quality in green. RMS values following AO correction ranged from 0.08 to 0.20, and the corresponding VS in green from 0.55 to 0.88 (nearly diffraction-limited in most subjects). The improvement in VS in green with AO correction was higher in subjects with lower optical quality than for subjects with higher optical quality: average area under the MTF increased for G_AO/G_NoAO from 3.11 to 5.25 (S#1–S#5) compared with only from 6.92 to 7.09 (S#6–S#10). The opposite occurred for the blue, subjects with poorer optics experienced a larger MTF in blue with natural aberrations than with AO-correction: MTF B_NoAO/B_AO: 2.42–1.34 (S#1–S#5) vs 0.92–0.73 (S#6–S#10).

### Optical quality and psychophysical scores

**Figure 3:**
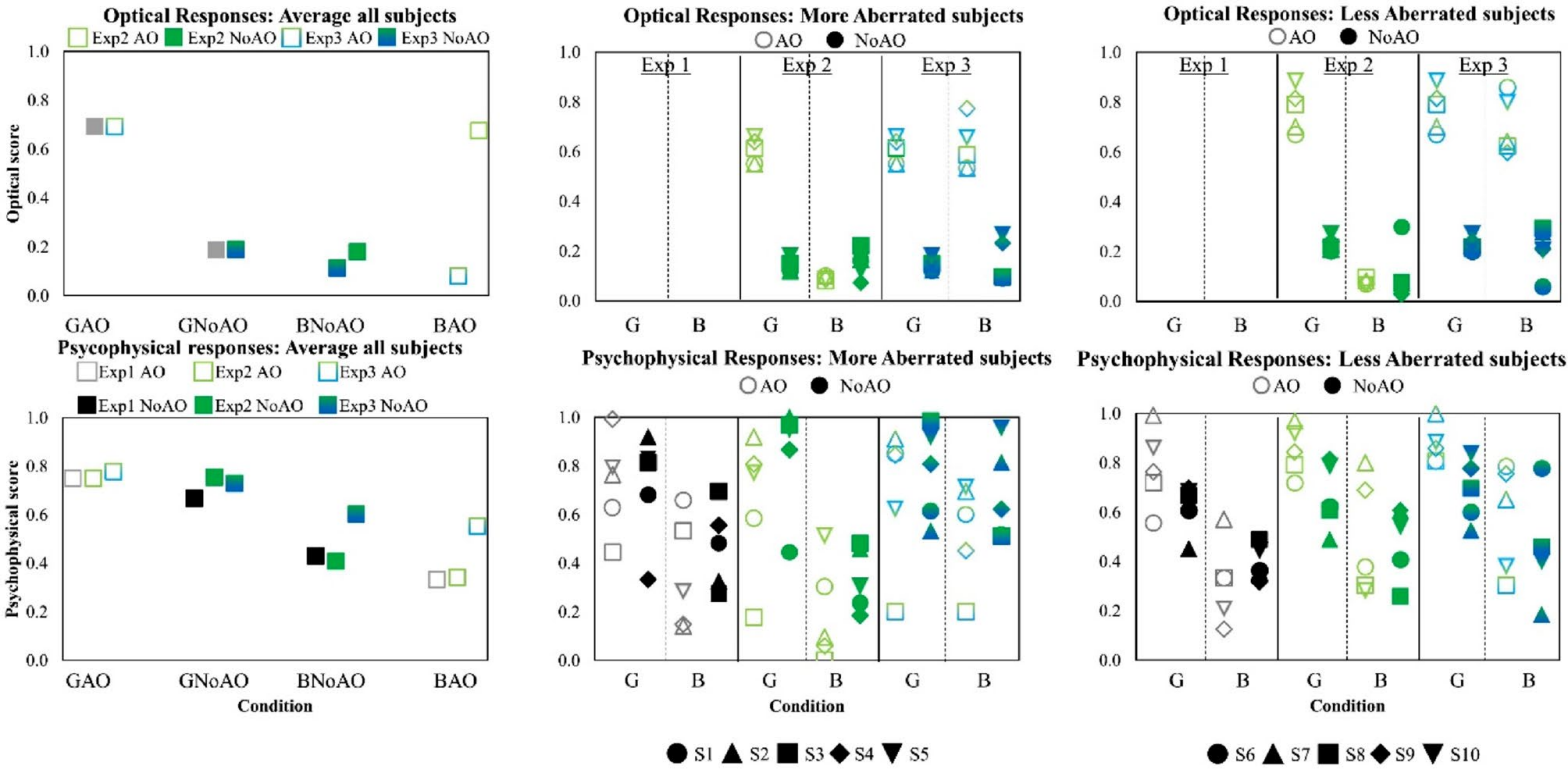
Results summary. Optical image quality (top panels) and Psychophysical scores (bottom panels). Left panels represent average data across all subjects. Middle panels represent data of individuals with more aberrations (S#1–S#5; VS < 0.2), and right panels data of individual less aberrations (S#6–S#10; VS > 0.2). Open bars represent results under corrected monochromatic aberrations (AO) and filled bars under natural aberrations (NoAO). Gray bars represent results for grayscale images (Exp 1), Green bars for green images (Exp 2) and graded green–blue bars for green and blue images (Exp 3). In the middle and right panels, open symbols stand for results under corrected monochromatic aberrations (AO) and closed symbols for results under natural aberrations (NoAO). Black symbols correspond to Exp 1, green symbols to Exp 2 and green and blue symbols to Exp 3.

Subjects scored^27^ the perceived quality of monochromatic grayscale and green images in focus and out of focus and blue images (for best focus in green), projected in a visual display viewed through Adaptive Optics. Figure 3 compares measured optical quality and psychophysical scores. Optical quality—top panels—are normalized area under the MTF (from Exp 2 and 3). The psychophysical scores in the bottom row of panels represent the normalized perceived image quality responses in the four evaluated conditions (G_AO, G_NoAO, B_AO and B_NoAO) for experiments 1, 2 and 3. For this scale, a value of 1.0 represents the highest perceived image quality. Left panels represent average data across subjects, and the middle and right panels represent data from individual subjects: S#1–S#5, (more aberrated, i.e. poorer optics; Middle) and S#6–S#10 (less aberrated, i.e. better optics; Right). The standard deviation of the perceptual score (0 to 5) across repetitions, averaged across conditions and experiments, is 0.882.

Optically (top panels), the effect of correcting natural aberrations in green (at best focus) is quite dramatic, with the optical quality (VS) improving on average from 0.19 (NoAO) to 0.69 (AO), i.e. they increase 2.2 × consistently in both Exp 2 and Exp 3. As expected, the relative improvement is larger for the more aberrated subjects (× 2.77, top middle panel, Exp 2 and 3) than for the less aberrated subjects (× 1.84, top right panel, Exp 2 and 3), due to the lower natural MTF in the more aberrated subjects.

On the other hand, correcting natural aberrations in grayscale or green images (in focus-bottom panels) had little impact on the perceptual scores (from 0.72 to 0.76, on average across the three experiments). Some subjects, particularly the more aberrated subjects (i.e., S1–S5) in fact judged image quality to be better without AO correction despite the large improvement in the MTF! The average perceptual score changed from 0.77 to 0.68 in the more aberrated subjects (middle) and from 0.67 to 0.83 on average in less aberrated subjects (right panel), upon correction of their natural aberrations.

For blue images, or green images defocused by an equivalent (blue) chromatic defocus, perceived image quality is consistently higher with natural aberrations (0.41 and 0.60, Exp 2 and 3, respectively) than with AO-correction (0.34 and 0.55, Exp 2 and 3, respectively). The improvement of the perceptual score with natural aberrations in blue (B_ NoAO/B_AO ratio) is larger in the more aberrated group. Interestingly, while the results are similar for the three perceptual experiments for gray or green images in focus, perceptually blue images (with inherent chromatic defocus) are judged consistently shaper than the gray or green images defocused by an equivalent amount of defocus (0.60 vs 0.43–0.41, under natural aberrations; 0.55 vs 0.33–0.34, under AO-correction). The improvement in perceived image quality of blue images, defocused by chromatic aberration, is largest in the more aberrated group.

Mixed two-way ANOVA analysis of differences across conditions indicated similarities across optical measurements and the three psychophysical experiments. Optically, a significant main effect (p < 0.001) of conditions (G_AO, G_NoAO, B_AO, B_NoAO) was found while psychophysically, both the conditions and the three experiments (different chromatic content) main effects were significant (p < 0.001, and 0.017, respectively). Also, optically, the quality for green stimuli for corrected aberrations (G_AO) is significantly higher (p < 0.05) than for natural aberrations (G_NoAO) for all subjects. On the other hand, the quality of blue stimuli (or green stimuli with equivalent chromatic defocus) for corrected aberrations (B_AO) is significantly lower (p < 0.05) than for natural aberrations (B_NoAO) in the group of subjects with lower optical quality (higher aberrations), which means that the described effect is stronger in subjects with poorer optics.

In green light in focus, optical predictions are highly positively correlated with psychophysical responses with natural aberrations (noAO), both in the more aberrated subjects (slope = 6.4; r = 0.45) and in the less aberrated subjects (slope = 3.9, r = 0.52), however the correlations are weak when aberrations are corrected (AO, slope = 0.13, r = 3·10^–4^ and slope = 0.13 r = 0.02, in the more and less aberrated groups respectively). The correlation between optical predictions and psychophysical responses is also highly positive in the more aberrated eyes with green light out of focus and AO correction (slope = 6.4, r = 0.16), however, it is negatively correlated or uncorrelated in the less aberrated subjects in green light out of focus (AO, slope = − 2.4, r = 0.01; and NoAO, slope = − 0.51, r = 0.14, respectively), and blue light (AO, slope = 0.22, r = 0.014; and NoAO, slope = − 1.9, r = 0.47). The lower slopes in the correlations (or no-correlation) between optical predictions and positive response, particularly natural conditions in both groups, is indicative of neural aspects (and not only optical degradation) playing a role in the perception of chromatic/out of focus targets.

### Control experiment using a polychromatic AO set-up

**Figure 4:**
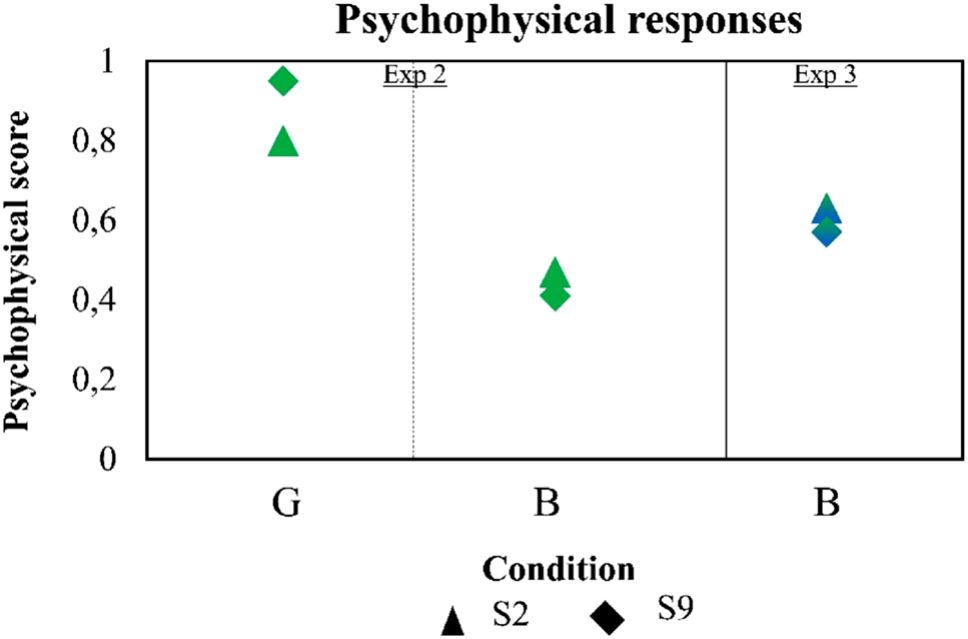
Results from the control experiment using narrow spectral bandwidth stimuli. Normalized psychophysical score of the perceived quality of natural images (555 nm for G, 480 nm for B, 5-nm spectral bandwidth), for experiments equivalent to Exp 2 and Exp 3. Exp 2 Green stands for green stimuli at best focus, Exp 2_B stands for green stimulus defocused by − 0.87 D with respect to the best focus in G, and Exp 3 Blue stands for blue stimulus at the best focus in G. Data are for two subjects (S2, S9).

In the control experiment one of the subjects from the more aberrated group (S2) and one from the less aberrated group (S9) repeated the experiment on the polychromatic AO system. As in the main experiment, the highest scores (0.88, on average) were found for the green stimulus in focus (G_NoAO). However, the scores for the defocused green images (equivalent to B_NoAO in Experiment 2) were lower (0.44, on average) than the score for blue images (B_NoAO in Experiment 3), 0.44 on average. Psychophysical scores for these two subjects with the narrow spectral band stimuli are shown in Fig. 4.

## Discussion

The normal focus for the eye is in the middle of the spectrum^31^ and under this condition, the interaction of the eye’s monochromatic and chromatic aberrations improve optical quality at short wavelengths. We found that the degree of improvement varied widely across individuals, with the largest positive interaction occurring for eyes with larger monochromatic aberrations (poorer optics). This occurs because the eye’s aberrations increase the eye’s depth-of-focus, improving optical quality for out-of-focus stimuli compared to the diffraction-limited eye (perfect optics). This increase in depth-of-focus is larger for those eyes with larger amounts of aberrations, and occurs similar to the way spherical aberration expands depth-of-focus in aberrated eyes. We performed simple optical calculations combining − 0.87 D of defocus (equivalent chromatic blur in blue) with positive spherical aberration (> + 0.15 µm, in 5-mm pupils) or with coma (> + 0.06 µm or < − 0.125 µm). These calculations produce an increased Visual Strehl for out of focus aberrated system compared to a defocused, otherwise diffraction limited system. For our two groups of subjects, the average spherical aberration was + 0.18 µm and + 0.08 µm, and the coma was 0.14 and 0.09, for the more and less aberrated group respectively. These results suggest that the presence of these aberrations plays a relevant role in the experimental results. Our findings replicate and expand on a previous publication^15^, which reported results on three eyes. Here we demonstrate that the effect is graded based on the naturally occurring aberrations.

Surprisingly, there is a pronounced difference in the corresponding perceptual response to these variations in image quality. This finding seems to arise from the ability of individuals to adapt to their own aberrations^2,23^. This adaptation causes individual variations in optical quality to be less perceptually important. Perceptual image quality scores tended to be higher for focused green images than for grayscale images, but only when the higher order monochromatic aberrations were corrected. This is consistent with a larger impact of LCA when monochromatic aberrations are corrected by AO. However, correcting high order monochromatic aberrations only improved perceived image quality by a factor of × 1.07, on average. This finding that a large improvement in optical quality leads only to small improvements psychophysically is consistent with prior work. For example a previous study reported an average increase in the MTF by × 8, whereas the CSF increased by only 1.35 times^32^ with AO-correction. An attenuated visual benefit of correcting the eye’s optics is not surprising given the neural adaptation to the subjects’ native aberrations^2^. While subjects generally identify images as sharper when viewed through AO-corrected optics^22^, previous work has shown that the amount of blur in the image that produces highest perceived image quality matches the level of blur (and blur orientation) produced by the native aberrations of their eyes^2,23^. The evidence that it is a neural adaptation is supported by the fact that the native blur does not produce aftereffects (while scaled versions of that blur do^2^). Also in experiments where subjects judged images blurred with different individual’s aberrations (from low to highly aberrations), the image judged as perceptually best corresponds to that blurred with the subject’s own aberrations (or similar amounts of blur)^2^.

Our results also reveal that, on average, out of focus blue images appear less blurred when the native aberrations are present than when they are corrected, in line with the optical findings^15^. However, perceived quality of blue images (when the eye is focused for green) is relatively high, either with or without high order monochromatic aberrations. And what is most relevant, blue images (naturally defocused by chromatic blur) are psychophysically judged as sharper than green (or monochromatic grayscale) images defocused by the same amount of equivalent blur (− 0.87 D). The higher perceived quality of defocused blue images compared to defocused green or luminance-contrast (grayscale) images suggests that the visual system is calibrated based on the average chromaticity, and due to the univariance of the cones, the mechanism of adaptation might use color information to discount perceptual blur or loss of sharpness.

A potential reason for reduced sensitivity to defocus in blue may be associated with the reported increased psychophysical depth-of-focus in blue, not caused by the optics^33^, as objective depth-of-focus does not vary across wavelengths^6^. The sparse sampling of S cones alone is unlikely to cause this effect. As the luminance contrast, determined by L and M cone signals, is similar for all three stimuli once the optical effect is accounted for. The spectral range of the blue images in the main experiment (CRT blue gun) strongly stimulates all cone types. This finding is consistent with the observation that the increase in perceived blur with increasing physical defocus is higher for luminance than for blue-yellow stimuli. This may explain the observation that compressing the color information in a natural scene (as done in JPEG compression^34^) produces little or no impression of blur, as opposed to a much higher blur sensitivity to changes in the luminance layer^35^. Although our stimuli are not based on chromatic contrast (green and blue stimuli provided luminance contrast and were matched in average luminance) higher spatial sensitivity for luminance than chromatic contrast variations may prevail in this effect. It is also important to note that the effect appears to be intrinsic to the dominant wavelength, and not biased by the spectral bandwidth of the images, as a control experiment showed similar results using stimuli illuminated with narrow spectral bandwidth (5 nm) blue and green light.

We conclude that the observed higher perceived quality of defocused blue stimuli is influenced by neural adaptation mechanisms. The shift in psychophysical score for blue (as compared to the same defocus in green) may underlie contingent adaptation to blue and out-of-focus images (as the blue component of images is normally out-of-focus). It also may suggest that observers are naturally adapted to both the blur produced by their native aberrations for stimuli where L and M cone signals are predominant (the middle of the spectrum) and to the effect produced by natural defocus in blue.

In summary, the presence of monochromatic optical aberrations protects vision against chromatic defocus. However there is also an equally important role for adaptational mechanisms which are involved in contrast constancy, and work differentially across wavelengths.
